# Supplementary material for: Efficacy of Penile Low-Intensity Shockwave Therapy and Determinants of Treatment Response in Taiwanese Patients with Erectile Dysfunction
Source: Biomedicines. 2021 Nov 12;9(11):1670. doi: 10.3390/biomedicines9111670 (PMC8615607; doi:10.3390/biomedicines9111670)
Supplement: Supplementary file 1 [file biomedicines-09-01670-s001.zip › ED_LiSWT_Suppl. Table S1.pdf]

Supplementary Table S1. The ED severity - stratified efficacy of Li-ESWT

ED severity / EF index	Mild (n = 9)	Mild-to-Moderate (n = 16)	Moderate (n = 20)	Severe (n = 24)
1st Month				
IIEF-5	1.67*	4.94***	6.50***	9.17***
EHS	0.55*	0.68**	0.75***	1.20***
Success* n (%)	4 (44.4%)	11 (68.8%)	11 (55%)	13 (54.2%)
QoL	-0.38	-0.82*	-0.24	-0.46
3rd Month				
IIEF-5	0.89	5.31***	6.35***	8.54***
EHS	0.55*	0.75***	0.80***	1.16***
Success* n (%)	2 (22.2%)	12 (75%)	10 (50%)	13 (54.2%)
QoL	-0.5	-1.0**	-0.47	-0.46
6th Month				
IIEF-5	1.00	4.56***	5.80***	7.80***
EHS	0.67**	0.81***	0.60**	1.16***
Success* n (%)	4 (44.4%)	10 (62.5%)	10 (50%)	14 (58.3%)
QoL	-0.625	-0.91**	-0.42*	-0.46
12th month				
IIEF-5	0.33	4.19**	4.80***	7.00***
EHS	0.67**	0.81***	0.55**	1.0***
Success* n (%)	4 (44.4%)	8 (50%)	8 (40%)	12 (50%)
QoL	-0.67	-1.0	-0.29	-0.59*

*success defined by MCID, minimal clinically important difference; ED, erectile dysfunction; EF, erectile function; Li-SWT, low intensity shockwave therapy; IIEF-5, 5-item international index of erectile function; EHS, erectile hardness score; QoL, quality of life; *$p<0.05$, **$p<0.01$, ***$p<0.001$
