# Supplementary material for: Efficacy of Penile Low-Intensity Shockwave Therapy and Determinants of Treatment Response in Taiwanese Patients with Erectile Dysfunction
Source: Biomedicines. 2021 Nov 12;9(11):1670. doi: 10.3390/biomedicines9111670 (PMC8615607; doi:10.3390/biomedicines9111670)
Supplement: Supplementary file 1 [file biomedicines-09-01670-s001.zip › ED_LiSWT_Suppl. Table S2.pdf]

Supplementary Table S2. The PDE5i response - stratified efficacy of Li-ESWT

	PDE5i responders (n = 17)	PDE5i non-responders (n = 52)	*p*-value
1st Month			
IIEF-5	7.06	6.23	0.66
EHS	0.70	0.92	0.36
Success* n (%)	10 (58.8%)	29 (55.7%)	0.85
QoL	-0.41	-0.46	0.91
3rd Month			
IIEF-5	6.71	6.10	0.69
EHS	0.64	0.96	0.17
Success* n (%)	10 (58.8%)	27 (51.9%)	0.62
QoL	-0.66	-0.54	0.71
6th Month			
IIEF-5	5.94	5.54	0.79
EHS	0.64	0.92	0.23
Success* n (%)	8 (47.1%)	30 (57.7%)	0.44
QoL	-0.58	-0.55	0.92
12th month			
IIEF-5	4.65	4.88	0.89
EHS	0.47	0.90	0.08
Success* n (%)	8 (47.1%)	24 (46%)	0.94
QoL	-0.75	0.55	0.56

*success defined by MCID, minimal clinically important difference; Li-SWT, low intensity shockwave therapy; 95%CI, 95% confidence interval; IIEF-5, 5-item international index of erectile function; EHS, erectile hardness score; QoL, quality of life
